# The Prevalence of Hypertension Among Children and Antihypertensive Use: Protocol for a Systematic Review and Meta-Analysis

**DOI:** 10.2196/65807

**Published:** 2025-05-12

**Authors:** Nur Hasnah Maamor, Nur Ain Zahidah Zainudin, Nur Liana Md Nasir, Kasturi Manoharan, Sharifah Zawani Syed Ahmad Yunus, Nor Asiah Muhamad, Lai Nai Ming

**Affiliations:** 1 Sector for Evidence-based Healthcare National Institutes of Health Shah Alam Malaysia; 2 Virology Unit, Infectious Disease Research Centre Institute for Medical Research National Institutes of Health Shah Alam Malaysia; 3 Toxicology and Pharmacology Unit, Herbal Medicine Research Centre Institute for Medical Research National Institutes of Health Shah Alam Malaysia; 4 Ethics & Research Surveillance Sector National Institutes of Health Shah Alam Malaysia; 5 Scientific Communication and Dissemination Unit National Institutes of Health Shah Alam Malaysia; 6 School of Medicine Taylor’s University Malaysia Subang Jaya Malaysia

**Keywords:** children hypertension, protocol, systematic review, meta-analysis, hypertension, children, anti-hypertensive, prevalence, childhood hypertension, risk factor, teenager, HPN, high blood pressure, blood pressure

## Abstract

**Background:**

Early-onset hypertension (HT) presents compounded risks for cardiovascular, renal, and other systemic complications. Childhood HT is associated with HT during adulthood and detrimental lifelong cardiovascular disease events. However, most of the cases are not detectable as HT measurement in children is complicated and unstable. The global prevalence of HT among children is rapidly increasing. A previous study (2019) reported that the pooled global HT prevalence is 4.0% and the number is believed to be elevated. However, prevalence estimates of childhood HT have rarely been synthesized globally.

**Objective:**

This study aims to systematically pool all evidence from published articles and synthesize the evidence on the global prevalence of childhood HT and antihypertensive (anti-HT) use among children and its effects.

**Methods:**

This systematic review of observational and experimental studies will investigate the overall prevalence of HT and anti-HT use among children. We will search articles from 4 web-based databases: PubMed, Cochrane Central Register of Controlled Trials (CENTRAL), Embase, and Scopus using the specific keywords across the databases. During the article search conducted until October 2024, we retrieved 14,575 articles. Articles published in the English language with full text and are peer-reviewed journals included children aged between 0 and 18 years, confirmed with HT or high blood pressure (BP) on at least 3 separate occasions, and stated the definition of HT will be included in this protocol. Study selection and reporting will follow the PRISMA (Preferred Reporting Items for Systematic Reviews and Meta-Analyses) and the Meta-Analysis of Observational Studies in Epidemiology guidelines and Cochrane Risk of bias tool (ACROBAT) for experimental studies. Data will be extracted using a standardized data extraction form using Microsoft Excel software and the studies’ quality will be assessed using the Joanna Briggs Institute’s guideline according to the study design. We will use STATA software (version 17.0; StataCorp LLC) to calculate the global pooled prevalence and RevMan software (version 5.4; StataCorp LLC) to observe the effect of anti-HT use and BP among children. The risk of bias will be assessed using a funnel plot.

**Results:**

We retrieved 14,575 articles from 4 databases in October 2024. We will report the current global overall or pooled prevalence of HT as well as by region, risk factors, anti-HT use, and the anti-HT BP-lowering effect among the general children population. The findings will be presented in summary table findings and forest plot. This review is expected to be completed in the middle of 2025.

**Conclusions:**

This review will provide a comprehensive synthesis of the overall prevalence of HT among children—a public health issue of growing concern with long-term impact. This review will also provide important information to inform practice in developing effective strategies for preventing and managing childhood HT.

**Trial Registration:**

PROSPERO CRD42024500248; https://www.crd.york.ac.uk/PROSPERO/view/CRD42024500248

**International Registered Report Identifier (IRRID):**

PRR1-10.2196/65807

## Introduction

### Overview

Hypertension (HT) occurs when the blood pressure (BP) is elevated above the normal range. It is a major risk factor for cardiovascular diseases (CVD), chronic kidney disease, and a major cause of death [1]. Studies have suggested HT in childhood to be associated with essential HT in adulthood [2,3]. However, childhood HT is less commonly studied and reported than adult HT [4] and receives less attention in public health [5]. Globally, confirmed HT is prevalent in 2%-4% of children [6]—as also demonstrated in the United States (2.2%-3.9%) [7]. Whereas in the African and Asian continents, the pooled prevalence of HT among children is 5.5% (Africa) [8], 18.4% (China) [9], and 29.8% (India) [10].

Evidence from pathophysiological and epidemiological studies suggests that essential HT and precursors of CVDs (atherosclerosis, stroke, left ventricular hypertrophy, and decreased cognitive function) [11], begin in childhood but often go unnoticed unless specifically monitored [3]. Genetics and lifestyle factors are associated with HT [12-14]. For example, high salt intake increases the risk of HT in young people [15] and high BMI during adolescence is associated with an increased risk of developing HT or CVD in adulthood [16].

Early intervention is important to reduce the burden and future complications of HT [1]. The effects of antihypertensive (anti-HT) from the classes of diuretics, beta blockers, angiotensin-converting enzyme inhibitors, angiotensin receptor blockers, calcium channel blockers, and vasodilators on children have been investigated [17-19]. However, there is scarce evidence on the association between anti-HT and the decrease of BP in children. Therefore, we will conduct a systematic review and meta-analysis to synthesize the evidence on the current global overall or pooled prevalence of HT, risk factors, anti-HT use, and the anti-HT BP-lowering effect among the general children population.

### Review Questions

1. What is the estimated pooled prevalence for children globally, and by regions (Asia, Africa, Europe, Oceania, North and South America)?
2. What is (are) the type of anti-HT medication(s) taken by children and the estimated decrease of the mean systolic BP and diastolic BP?
3. What are the risk factors associated with HT among children?

## Methods

### Overview

This protocol is registered with the International Prospective Register of Systematic Review (PROSPERO) and the review will be conducted and reported following the PRISMA (Preferred Reporting Items for Systematic Reviews and Meta-analysis) protocol [20].

### Study Design

We will include all empirical study designs including observational (eg, case control, cohort, and cross-sectional) and experimental studies (randomized controlled trial [RCT], non-RCT, and cluster RCT) to capture all desired outcomes. No restriction will be imposed on the date of publication and sample size.

### Study Area

We will include all published studies with information on HT among children globally and divide the studies according to the regions.

### Type of the Participants

We will include primary studies that investigated children (0 to 18 years old) that provide estimates of any stage of systemic HT, prehypertension, or systolic or diastolic HT. If we find studies that were conducted for both adults and children, we will separate the prevalence data of HT for children (0-9 years) or adolescents (10-18 years) following the World Health Organization classification [21]. If the data is not amenable to the World Health Organization classification, we will contact the author to obtain clarification/data in studies.

We will include studies that explicitly describe the methods of measuring BP, definitions of HT, initial systolic BP and diastolic BP, posttreatment systolic BP and diastolic BP, anti-HT medication used, duration of treatment, dosage, sample size, number of individuals with HT, prevalence of HT, and others. Studies with BP measurements with a minimum of 3 separate readings are also eligible to be included. We will exclude studies on the adult population (aged 19 years and older), or any population with abnormal groups or conditions such as obese and disabled children or children with specific diseases. We will also exclude articles that do not explain the definition of HT, include at least 3 BP measurements, are not in English, or whose full text is unattainable. Editorials, review articles, letters to the editor, abstracts and poster presentations will also be excluded from this review. If we find multiple articles with the same data, then we will use the study with the largest sample size. Findings will be presented in tables and figures.

### Type of Outcome Measures

The primary outcome will be the pool prevalence of HT among children. We will measure the pool prevalence according to different age groups such as younger than 7 years, between 7 and 12 years, and older than 12 years to 18 years, if the data are available. Secondary outcomes will be HT associated with morbidity such as stroke, coronary heart disease, diabetes, or end-stage renal disease; types of anti-HT intake; the decrease of systolic BP and diastolic BP number after anti-HT intake; and the effect of anti-HT on the BP.

### Searching Methods for Identification of Studies

#### Electronic Search

We will search using 4 databases—PubMed, Cochrane Central Register of Controlled Trials (CENTRAL), Embase, and Scopus—to identify eligible studies. There is no restriction on the publication date, however, the language will be restricted to English. The search will be performed up to October 2024.

#### Search Strategy

The proposed search term for the first theme will be “hypertension”—which includes high BP or HT or HTN or HBP. The second theme is “children” including adolescents (aged 10-18 years) or neonates. The third theme is “antihypertensive medication,” which includes all classes of anti-HT medication, and the other similar name used. The PICO (Population, Intervention, Comparison, and Outcomes) framework will be used during the article search; “P (population)” refers to children, and “O (outcome)” refers to risk factors, anti-HT medications taken to estimate the decrease of the mean systolic BP and diastolic BP. Both “I (Intervention)” and C “(comparisons)” aspect is not included in this review. Table 1 lists the search strategy using PICO framework. The exploded versions of MeSH (Medical Subject Headings) will be used for the first theme. All themes will be searched for using “AND” in combination. The details for the proposed search terms for all the databases are listed in Textbox 1.

**Table 1:** Search strategy using the PICO (Population, Intervention, Comparison, and Outcomes) frameworks.

PICO elements	Keywords
Population	Children aged 0-18 years with hypertension
Intervention	N/A^a^
Comparison	N/A
Outcomes	Prevalence, risk factor, anti-HT^b^, and the decrease of BP^c^

^a^Not applicable.

^b^anti-HT: antihypertensive.

^c^BP: blood pressure.

**Proposed search terms.**

**PubMed**

Search: Hypertension OR HTN OR HT [MeSH Terms]

AND

Child*[Title/Abstract]) OR (Adolescent*[Title/Abstract] OR (Neonate*[Title/Abstract] OR (Teenager*[Title/Abstract] OR (Toddler*[Title/Abstract] OR (Newborn*[Title/Abstract] OR (Infant*[Title/Abstract] OR (Youth*[Title/Abstract] OR (Boy [Title/Abstract] OR (Boys[Title/Abstract] OR (Girl*[Title/Abstract] OR (Youngster*[Title/Abstract]

AND

Hypertension [Title/Abstract] OR (High blood pressure [Title/Abstract] OR (Elevated blood pressure [Title/Abstract]) OR (HT[Title/Abstract] OR (HBP[Title/Abstract] OR (HTN[Title/Abstract]

AND

Prevalence [Title/Abstract] OR (Epidemiology [Title/Abstract] OR (Population [Title/Abstract] OR (Blood pressure [Title/Abstract]

AND

Antihypertensive medication[Title/Abstract] OR (Antihypertensive drug[Title/Abstract] OR (Anti-HTN[Title/Abstract] OR (Anti-HT[Title/Abstract] OR (Diuretics[Title/Abstract]) OR (Thiazide[Title/Abstract]OR (Hydrochlorothiazide[Title/Abstract] OR (Bendroflumethiazide[Title/Abstract] OR (Loop[Title/Abstract]) OR (Furosemide[Title/Abstract] OR (Bumetanide[Title/Abstract] OR (Torsemide[Title/Abstract] OR (Potassium sparing[Title/Abstract] OR (Distal, potassium retaining[Title/Abstract] OR (Spironolactone[Title/Abstract] OR (Eplerenone[Title/Abstract] OR (Combination diuretics[Title/Abstract] OR (Spironolactone + hydrochlorothiazide[Title/Abstract] OR (Spironolactone and hydrochlorothiazide[Title/Abstract] OR [Beta-blockers[Title/Abstract]) OR (Propranolol[Title/Abstract] OR (Inderal[Title/Abstract] OR (Atenolol[Title/Abstract] OR (Metoprolol tartrate[Title/Abstract] OR (Metoprolol succinate[Title/Abstract] OR (Labetalol[Title/Abstract] OR (Bisoprolol[Title/Abstract] OR (Carvedilol phosphate[Title/Abstract] OR (Combination beta-blocker/diuretic[Title/Abstract] OR (Combination beta-blocker and diuretic[Title/Abstract] OR (Hydrochlorothiazide[Title/Abstract] and bisoprolol[Title/Abstract] OR (Hydrochlorothiazide + bisoprolol[Title/Abstract] OR ACE inhibitors[Title/Abstract] OR (Angiotensin Converting Enzyme Inhibitors[Title/Abstract] OR (ACEI[Title/Abstract] OR (Benazepril[Title/Abstract] OR (Captopril[Title/Abstract] OR (Enalapril[Title/Abstract] OR (Fosinopril [Title/Abstract] OR (Lisinopril[Title/Abstract] OR (Moexipril[Title/Abstract] OR (Quinapril[Title/Abstract] OR (Ramipril[Title/Abstract] OR (Perindopril[Title/Abstract] OR (ARB[Title/Abstract] OR (Angiotensin receptor blockers[Title/Abstract] OR (Angiotensin II receptor blockers[Title/Abstract] OR (Candesartan[Title/Abstract] OR (Irbesartan[Title/Abstract] OR (losartan[Title/Abstract] OR (Olmesartan[Title/Abstract] OR (Telmisartan[Title/Abstract] OR (Valsartan[Title/Abstract]OR (Calcium channel blockers (CCB[Title/Abstract]OR (Calcium channel blockers[Title/Abstract] OR (Calcium channel antagonists[Title/Abstract])OR (Dihydropyridine group[Title/Abstract] OR (Amlodipine[Title/Abstract] OR (Felodipine[Title/Abstract])) OR (Nifedipine[Title/Abstract] OR (Nifedipine LA[Title/Abstract] OR (Nicardipine[Title/Abstract] OR (Isradipine[Title/Abstract] OR (Clonidine[Title/Abstract] OR ((Nondihydropyridine group[Title/Abstract] OR (Verapamil[Title/Abstract] OR (Alpha blockers[Title/Abstract] OR (Doxazosin[Title/Abstract]OR (Prazosin[Title/Abstract] OR (Terazosin hydrochloride[Title/Abstract] OR (Alpha-2 receptor agonists[Title/Abstract] OR (Methyldopa[Title/Abstract] OR (Guanfacine[Title/Abstract] OR (Vasodilators[Title/Abstract] OR (Hydralazine[Title/Abstract]OR (Minoxidil[Title/Abstract]

**Scopus**

TITLE-ABS-KEY (Child*OR Adolescent* OR Neonate*OR Teenager* OR Toddler*OR Newborn* OR Infant*OR Youth* OR Boy OR Boys OR Girl* OR Youngster*)

AND

TITLE-ABS-KEY (Hypertension OR “High blood pressure” OR “Elevated blood pressure” OR HT OR HBP OR HTN)

AND

TITLE-ABS-KEY (“Antihypertensive medication” OR “Antihypertensive drug” OR “Anti-HTN” OR “Anti-HT” OR Diuretics OR Thiazide OR Hydrochlorothiazide OR Bendroflumethiazide OR Loop OR Furosemide OR Bumetanide OR Torsemide OR “Potassium sparing” OR Distal OR “potassium retaining” OR Spironolactone OR Eplerenone OR “Combination diuretics” OR “Spironolactone + hydrochlorothiazide” OR “Spironolactone and hydrochlorothiazide” OR “Beta-blockers” OR Propranolol OR Inderal OR Atenolol OR “Metoprolol tartrate” OR “Metoprolol succinate” OR Labetalol OR Bisoprolol OR “Carvedilol phosphate” OR “Combination beta-blocker/diuretic” OR “Combination beta-blocker and diuretic” OR “Hydrochlorothiazide and bisoprolol” OR “Hydrochlorothiazide + bisoprolol” OR “ACE inhibitors” OR “Angiotensin Converting Enzyme Inhibitors” OR ACEI OR Benazepril OR Captopril OR Enalapril OR Fosinopril OR Lisinopril OR Moexipril OR Quinapril OR Ramipril OR Perindopril OR “Angiotensin receptor blockers” OR ARB OR “Angiotensin II receptor blockers” OR Candesartan OR Irbesartan OR losartan OR Olmesartan OR Telmisartan OR Valsartan OR “Calcium channel blockers” OR CCB OR “Calcium channel antagonists” OR “Dihydropyridine group” OR Amlodipine OR Felodipine OR Nifedipine OR “Nifedipine LA”OR Nicardipine OR Isradipine OR Clonidine OR “Nondihydropyridine group” OR Verapamil OR “Alpha blockers” OR Doxazosin OR Prazosin OR “Terazosin hydrochloride” OR “Alpha-2 receptor agonists” OR Methyldopa OR Guanfacine OR Vasodilators OR Hydralazine OR Minoxidil

**CENTRAL (Cochrane Central Register of Controlled Trials)**

MeSH descriptor: [Hypertension OR HTN OR HT] explode all trees

AND

Search: Hypertension OR HTN OR HT [MeSH Terms]

AND

Child*[Title/Abstract]) OR (Adolescent*[Title/Abstract])) OR (Neonate*[Title/Abstract])) OR (Teenager*[Title/Abstract])) OR (Toddler*[Title/Abstract])) OR (Newborn*[Title/Abstract])) OR (Infant*[Title/Abstract])) OR (Youth*[Title/Abstract])) OR (Boy[Title/Abstract])) OR (Boys[Title/Abstract])) OR (Girl*[Title/Abstract])) OR (Youngster*[Title/Abstract]))

AND

Hypertension [Title/Abstract] OR (High blood pressure [Title/Abstract] OR (Elevated blood pressure [Title/Abstract]) OR (HT[Title/Abstract] OR (HBP[Title/Abstract] OR (HTN[Title/Abstract]

AND

Prevalence [Title/Abstract] OR (Epidemiology [Title/Abstract] OR (Population [Title/Abstract] OR (Blood pressure [Title/Abstract]

AND

Antihypertensive medication[Title/Abstract] OR (Antihypertensive drug[Title/Abstract] OR (Anti-HTN[Title/Abstract] OR (Anti-HT[Title/Abstract] OR (Diuretics[Title/Abstract]) OR (Thiazide[Title/Abstract]OR (Hydrochlorothiazide[Title/Abstract] OR (Bendroflumethiazide[Title/Abstract] OR (Loop[Title/Abstract]) OR (Furosemide[Title/Abstract] OR (Bumetanide[Title/Abstract] OR (Torsemide[Title/Abstract] OR (Potassium sparing[Title/Abstract] OR (Distal, potassium retaining[Title/Abstract] OR (Spironolactone[Title/Abstract] OR (Eplerenone[Title/Abstract] OR (Combination diuretics[Title/Abstract] OR (Spironolactone + hydrochlorothiazide[Title/Abstract] OR (Spironolactone[Title/Abstract] and hydrochlorothiazide[Title/Abstract] OR [Beta-blockers[Title/Abstract]) OR (Propranolol[Title/Abstract] OR (Inderal[Title/Abstract] OR (Atenolol[Title/Abstract] OR (Metoprolol tartrate[Title/Abstract] OR (Metoprolol succinate[Title/Abstract] OR (Labetalol[Title/Abstract] OR (Bisoprolol[Title/Abstract] OR (Carvedilol phosphate[Title/Abstract] OR (Combination beta-blocker/diuretic[Title/Abstract] OR (Combination beta-blocker[Title/Abstract] and diuretic[Title/Abstract] OR (Hydrochlorothiazide[Title/Abstract] and bisoprolol[Title/Abstract] OR (Hydrochlorothiazide + bisoprolol[Title/Abstract] OR ACE inhibitors[Title/Abstract] OR (Angiotensin Converting Enzyme Inhibitors[Title/Abstract] OR (ACEI[Title/Abstract] OR (Benazepril[Title/Abstract] OR (Captopril[Title/Abstract] OR (Enalapril[Title/Abstract] OR (Fosinopril[Title/Abstract] OR (Lisinopril[Title/Abstract] OR (Moexipril[Title/Abstract] OR (Quinapril[Title/Abstract] OR (Ramipril[Title/Abstract] OR (Perindopril[Title/Abstract] OR (ARB [Title/Abstract] OR (Angiotensin receptor blockers[Title/Abstract] OR (Angiotensin II receptor blockers[Title/Abstract] OR (Candesartan[Title/Abstract] OR (Irbesartan[Title/Abstract] OR (losartan[Title/Abstract] OR (Olmesartan[Title/Abstract] OR (Telmisartan[Title/Abstract] OR (Valsartan[Title/Abstract]OR (Calcium channel blockers (CCB[Title/Abstract]OR (Calcium channel blockers[Title/Abstract] OR (Calcium channel antagonists[Title/Abstract])OR (Dihydropyridine group[Title/Abstract] OR (Amlodipine[Title/Abstract] OR (Felodipine[Title/Abstract])) OR (Nifedipine[Title/Abstract] OR (Nifedipine LA[Title/Abstract] OR (Nicardipine[Title/Abstract] OR (Isradipine[Title/Abstract] OR (Clonidine[Title/Abstract] OR ((Nondihydropyridine group[Title/Abstract] OR (Verapamil[Title/Abstract] OR (Alpha blockers[Title/Abstract] OR (Doxazosin[Title/Abstract]OR (Prazosin[Title/Abstract] OR (Terazosin hydrochloride[Title/Abstract] OR (Alpha-2 receptor agonists[Title/Abstract] OR (Methyldopa[Title/Abstract] OR (Guanfacine[Title/Abstract] OR (Vasodilators[Title/Abstract] OR (Hydralazine[Title/Abstract]OR (Minoxidil[Title/Abstract]

**Embase**

(Antihypertensive medication [Title/Abstract] OR (Antihypertensive drug [Title/Abstract] OR (Anti-Ht [Title/Abstract] OR (Anti-HT[Title/Abstract] OR (diuretic [Title/Abstract] OR (thiazide[Title/Abstract] OR (hydrochlorothiazide[Title/Abstract] OR (Bendroflumethiazide[Title/Abstract] OR (Loop[Title/Abstract] OR (Furosemide[Title/Abstract] OR (Bumetanide[Title/Abstract] OR (Torsemide[Title/Abstract] OR (Potassium sparing[Title/Abstract] OR (Spironolactone[Title/Abstract] OR (Eplerenone[Title/Abstract] OR (Beta-blocker[Title/Abstract] OR (Propranolol[Title/Abstract] OR (Inderal[Title/Abstract] OR (Atenolol[Title/Abstract] OR (Metoprolol Tartrate[Title/Abstract] OR (Metoprolol Succinate[Title/Abstract] OR (ACE inhibitors[Title/Abstract] OR (Angiotensin converting enzyme inhibitor[Title/Abstract] OR (ACEI[Title/Abstract] OR (Benazepril[Title/Abstract] OR (Captopril[Title/Abstract] OR (Enalapril[Title/Abstract] OR (Fosinopril[Title/Abstract] OR (Lisinopril[Title/Abstract] OR (Moexipril[Title/Abstract] OR (Quinapril[Title/Abstract] OR (Ramipril[Title/Abstract] OR (Perindopril[Title/Abstract] OR (Angiotensin receptor blocker[Title/Abstract] OR (ARB[Title/Abstract] OR (Angiotensin II receptor blocker[Title/Abstract] OR (Candesartan[Title/Abstract] OR (Irbesartan[Title/Abstract] OR (Losartan[Title/Abstract] OR (Olmesartan[Title/Abstract] OR (Telmisartan[Title/Abstract] OR (Valsartan[Title/Abstract] OR (Calcium Channel Blockers[Title/Abstract] OR (CCB[Title/Abstract] OR (Calcium channel antagonist[Title/Abstract] OR (Dihydropyridine group[Title/Abstract] OR (Amlodipine[Title/Abstract] OR (Felodipine[Title/Abstract] OR (Nifedipine[Title/Abstract] OR (Nicardipine[Title/Abstract] OR (Isradipine[Title/Abstract] OR (Clonidipine[Title/Abstract] OR (Non dihydropyridine group[Title/Abstract] OR (Verampil[Title/Abstract] OR (Alpha blockers[Title/Abstract] OR (Doxazosin[Title/Abstract] OR (Terazosin hydrochoride[Title/Abstract] OR (Methyldopa[Title/Abstract] OR (Guanfacine[Title/Abstract] OR (Vasodilators[Title/Abstract] OR (Hydralazine[Title/Abstract] OR (Minoxidil[Title/Abstract]

AND

(Child*[Title/Abstract] OR (Adolescent*[Title/Abstract] OR (Neonate*[Title/Abstract] OR (Teenager*[Title/Abstract] OR (Toddler*[Title/Abstract] OR (Newborn*[Title/Abstract] OR (Infant*[Title/Abstract] OR (Youth*[Title/Abstract] OR (Boy*[Title/Abstract] OR (Girl*[Title/Abstract] OR (Youngster*[Title/Abstract]

AND

(Hypertension[Title/Abstract] OR (High blood pressure[Title/Abstract] OR (Elevated blood pressure[Title/Abstract OR (HTN[Title/Abstract]] OR (HT[Title/Abstract] OR (HBP[Title/Abstract]

AND

(Prevalence [Title/Abstract] OR (Blood pressure [Title/Abstract] OR (Risk factor*[Title/Abstract]

### Data Collection and Analysis

#### Selection of Studies

Two review authors (NAZZ and NLMN) will independently screen for titles and abstracts to identify the potential studies to be included. The studies will be identified and coded as “retrieve” (studies to be included, with potential to be included, or unclear) or “do not retrieve” (studies to be excluded). We will also identify and exclude duplicate studies. Two authors (KM and SZSAY) will retrieve the full text and review them against the inclusion and exclusion criteria along with the justifications. We will resolve any disagreement between the 2 reviewers via discussion or rechecking the full text. If a consensus cannot be reached, a third author (NHM) will deliberate.

**Figure 1:**
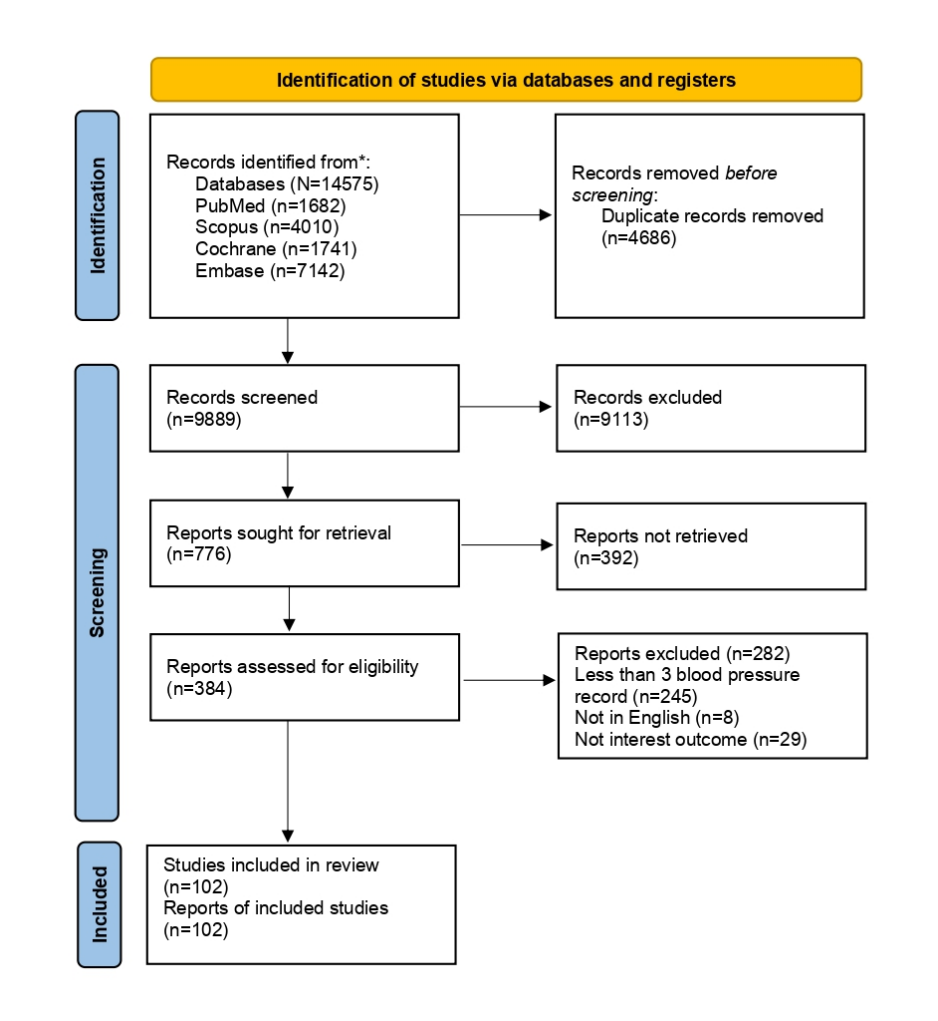
PRISMA flow chart.

We will record the selection process in sufficient detail to complete a PRISMA flow diagram [20,22] as shown in Figure 1. This review will use The Mendeley Reference Management Software [23] to store, arrange, and manage all articles identified from the databases.

#### Data Extraction and Management

We will use a standardized data extraction created in the Online Microsoft Excel Spreadsheet for study characteristics and outcome data to capture comprehensive information pertinent to the research question. Two review authors (KM and SZSAY) will independently extract the data from the included studies. If there is any disagreement on the data extraction, both authors will discuss and consult with 2 other authors (NAM and LLM). We will extract the following study characteristics from the included studies:

- Authors, year, title, country, region
- Participants: Total participants, HT individuals, age, sex, BMI, wrist and waist circumference, and risk factor for HT (such as family history or disease)
- Methods: Study design
- Analysis: Method used to measure BP reading, how many BP reading is repeated (measure at least 3 separate occasions), type of anti-HT used, the SBP and DBP reading after consumption of anti-HT, whether the patient also had another disease or comorbidities.

If there is any unclear data from the studies, we will validate it by contacting the corresponding author to provide any unclear, missing, or additional details.

#### Quality Assessment

The study’s quality will be evaluated using the Joanna Briggs Institute’s checklist by 3 review authors (NHM, NAZZ, and NLMN) independently. Any discrepancies in the assessment will be addressed through discussion among reviewers until a consensus is reached. Numerous Joanna Briggs Institute scales will be used in this review based on the type of study design (either cross-sectional, case-control, or cohort study). If we find experimental studies, we will use the ACROBAT NSI or Cochrane risk of bias tool to judge the study quality. The overall appraisal of the articles which are the potential to be included will be defined as either to include, exclude, or seek further info [24].

### Statistical Analysis

Data will be analyzed using STATA (version 17.0; StataCorp LLC) and RevMan software (StataCorp LLC). Statistical analysis will be divided into regions, age (preschool [less than 6 years old], elementary [7-12 years], and adolescent [13-18 years old]), device, and BMI. We will perform descriptive analysis and calculate the overall pool prevalence of HT among children using a random effect model to allow heterogeneity across the included studies and later divide the studies into regions. Freeman-Turkey transformation (arcsine square root transformation) will be applied to overcome the limitation of meta-analysis in prevalence studies as some of the included articles will have very low or very high prevalence. Having a wide range of prevalence values can result in disproportionately high weight due to the significant decrease in their inferred variance towards 0 [25]. The Freeman-Turkey transformation will be used for pooling to obtain a synthesized point estimate of prevalence with a 95% CI [26].

In addition to reporting the use of anti-HT medication, dosage, and duration of taking anti-HT, we will also document any other comorbidities or complications experienced by the patients. The mean and SD of BP readings (systolic BP and diastolic BP) before and after taking anti-HT medication will be recorded and analyzed in the RevMan software. We will also perform a meta-analysis to identify the risks and measure the effect of the intervention. A forest plot will be created for each pool estimate, and the distribution will be presented graphically. We plan to create a funnel plot if 10 or more studies are available to pool in a meta-analysis to assess reporting biases. We will assess funnel plot asymmetry visually. We will use a linear regression test to analyze the degree of publication bias with a *P* value of <.10 indicating significance for this test [27,28]. If the funnel plot is asymmetrical, (caused by a relationship between the effect size and sample size, or by publication bias), we will examine any observed effect for clinical heterogeneity [29]. Besides the funnel plot, we will also apply the Egger test to assess potential publication bias in meta-analysis [30].

We will conduct a meta-analysis by pooling the appropriate data using Cochrane’s statistical software, considering studies to be sufficiently similar in terms of population category, intervention, comparison, and outcome [31]. We will use a random effect model for pooled data. We will consider not pooling data if we encounter considerable heterogeneity (*I*^2^ of 75% or more) across studies. We will follow the strategies in the Cochrane Handbook for Systematic Reviews of Interventions for data management [27,28]. For multiple-arm studies, we will adjust the data following the methods described in the Cochrane Handbook for Systematic Reviews of Interventions [27,28]. For 2 arms that are relevant, we will include the relevant arms. If there are more than 2 relevant arms (eg, A vs control), we will set up separate pair-wise comparisons.

### Assessment of Heterogeneity

We will visually inspect the forest plots for any evidence of heterogeneity. We will assess heterogeneity using both Q test and *I*^2^ statistics. The *I*^2^ statistic will be used to quantify the impact of heterogeneity while Q test will be used to identify whether there is a significant heterogeneity occurring in the included studies. We will set the significance level for the Q test at 0.01 [32] while the *I*^2^ level of 75% indicates a substantial degree of heterogeneity. We will adopt a higher threshold of *I*^2^ in this due to heterogeneity inherent in the possible included article.

If the *I*^2^ of the overall studies is below 75%, the random effect model will be applied in the meta-analysis to overcome the inherent heterogeneity that might be highly present among different prevalent studies [33]. If significant heterogeneity is detected using the *I*^2^ index (>75%), we will investigate potential sources of heterogeneity using subgroup analyses and meta-regression. Subgroup analysis will be performed for the different study designs, gender, age, region, and specific risk factors using STATA software (version 17.0). Meta-regression will be performed using the same software. The possible causes will be explored and evaluated for their methodological characteristics to determine whether the degree of heterogeneity can be explained by differences in those characteristics and if a meta-analysis is appropriate. The overall prevalence and 95% CI estimate of individual studies will be presented in forest plots [4,34].

### Dissemination

This review will target a specific scientific publication targeting children as the population in their journals such as *The Journal of Pediatrics*, *Pediatrics*, *Journal of Child Health Care*, *Clinical Pediatrics*, and others. We also plan to publish in a high-impact journal that is suitable for our topic such as BMJ, PLOS, and JMIR. Few findings will be presented in the conferences both local and international as well as in any pediatric conference event. The preliminary findings will be presented to the Pediatric Clinical Practice, Ministry of Health Malaysia. This review will also help the clinicians to decide the best treatment for the patients and might be used as a reference during making documents for clinical practice guidelines for HT in children. We will also produce plain language summaries that will be shared with the public such as social media and health care websites.

### Ethical Considerations

We have registered this systematic review with the National Medical Research Register, Ministry of Health Malaysia (National Medical Research Register ID-24-00078-A7A). Since this review will only include published articles, ethical approval is not needed. This protocol has been registered with PROSPERO (CRD42024500248).

## Results

Article search will be performed in October 2024 and any articles published from inception until October 2024 will be included in this study before abstract and title screening. A total of 14,575 articles were retrieved from the 4 databases and the remaining 9889 articles were remaining for the title and abstract screening. About 776 articles are sought for retrieval and 384 articles will be screened for data extraction and eligibility (Figure 1). The findings will be visually presented in a summary of findings and a forest plot. The review is expected to be complete and published by the middle of 2025. Results on the global overall or pooled prevalence of HT, risk factors, anti-HT use, and the anti-HT BP-lowering effect among the general children population will be presented in this manuscript. Analysis for observational studies such as cohort, case-control, and cross-sectional and experimental studies will be analyzed separately.

## Discussion

### Principal Findings

The increased prevalence of HT among children between 2% and 5% contributed to significant challenges to public health and the economy [35]. Additionally, a previous review conducted in 2019 reported a global prevalence of HT in children at 4.0% [34], while the most recent study on BP and anti-HT was reported in 2018 [36,37]. It shows that the prevalence of HT among children is increasing yearly. Additionally, to the best of our knowledge, there is no available review on the prevalence, anti-HT, and decrease of the BP and risk factors among children were discussed in one paper. The strength of this review is rooted in the broad scope undertaken in HT among children. Therefore, the aim of this review is to highlight the updated overall prevalence of HT among children globally, drug therapy prescribed to manage HT, the reduction of BP following anti-HT regimens, and associated risk factors for HT among children. Additionally, among recent reviews, diagnostic criteria and therapeutic options are indicated for different medical conditions and comorbidities [38]. This review will conduct a qualitative analysis that may offer a deeper understanding of aspects that quantitative data alone may not capture [39]. Other studies also highlighted the necessity to revise current guidelines, to include the management of children with HT [40]. Despite evidence of HT prevalence in the general pediatric population [34], as stated previously, there is currently no updated systematic review and meta-analysis that simultaneously investigates the prevalence of HT and the effectiveness of anti-HT medication in decreasing BP in children.

This review will discuss the risk factors contributing to HT, such as BMI and family history. An increase in BMI in children has been associated with a higher risk of developing HT [41]. In China, the incidence of HT is higher in overweight and obese children compared with those with normal weight, with rates of 24.3% and 18.5% versus 11.1%, respectively [42]. Additionally, children with consistently high BMI trajectories are at greater risk for developing HT in later life [43].

While rapid weight gain during early childhood may predict higher BP, it may also increase the risk for cardiovascular risks during adulthood [44]. Similar findings have been reported in preterm births and low birth weight babies [45]. Children with asthma may also have an increased risk of HT [46] due to the interplay between asthma and HT likely involving inflammatory pathways. Furthermore, changes in vascular and asthma medications have been associated with increased BP, potentially contributing to HT [47].

Having a family history of HT also significantly influences the risk of HT among children, as there is a hereditary component to BP regulation [48]. Interestingly, exclusive breastfeeding has been linked to a decreased risk of HT in children. A study in China revealed that children who were exclusively breastfed had a reduction of 0.07 mm Hg in systolic BP and 0.05 mm Hg in diastolic BP [49].

We believe that our review will benefit both clinicians and researchers, especially in controlling BP among children as HT in childhood is a predictor of HT in adulthood that leads to elevated heart disease morbidity [50]. As the current cost of HT treatment is quite expensive, this review will also benefit in cost-effectiveness for HT treatment among children in the future. Thus, the findings from this review will inform clinical practice, particularly in prescribing anti-HT medications, and explore the most effective anti-HT treatment that can be applied to treat HT among children. This review will also be useful for both the local governments and policy makers to improve public awareness of HT among children, especially for the benefit of parents and caretakers.

### Potential Limitations of Review Methods

This review may have possible limitations due to the language restriction to include studies in English. This review will focus on the anti-HT among children and therefore, nonpharmacological interventions or prevention strategies will be excluded. The article search will be conducted using electronic databases and we will exclude the unpublished literatures which may potentially result in the oversight of some studies. Some resources may complicate the systematic retrieval as it potentially affects the overall reliability.

### Conclusions

This review will provide a comprehensive synthesis of the overall prevalence of HT among children a public health issue of growing concern with long-term impact. This review will also provide important information to inform practice in developing effective strategies for preventing and managing childhood HT.

## Data Availability

The datasets generated or analyzed during this study are available upon request from the corresponding author.

## Appendix

Multimedia Appendix 1 PRISMA-P (Preferred Reporting Items for Systematic Review and Meta-Analysis-Protocols) checklist.

## Abbreviations

anti-HT: antihypertensive
BP: blood pressure
CENTRAL: Cochrane Central Register of Controlled Trials
CVD: cardiovascular disease
HT: hypertension
MeSH: Medical Subject Headings
PICO: Population, Intervention, Comparison, and Outcomes
PRISMA: Preferred Reporting Items for Systematic Reviews and Meta-Analyses
PROSPERO: International Prospective Register of Systematic Review
RCT: randomized controlled trial
